# Problems or prospects? Being a parent in the early phase of the COVID-19 pandemic in Germany

**DOI:** 10.3389/fpsyg.2022.901249

**Published:** 2022-08-05

**Authors:** Angelika Ecker, Irina Jarvers, Daniel Schleicher, Stephanie Kandsperger, Iris Schelhorn, Marie Meyer, Thomas Borchert, Michael Lüdtke, Youssef Shiban

**Affiliations:** ^1^Clinic of Child and Adolescent Psychiatry, Psychosomatics and Psychotherapy, University of Regensburg, Regensburg, Germany; ^2^Department of Experimental Psychology, University of Regensburg, Regensburg, Germany; ^3^Department of Psychology, Private University of Applied Sciences, Göttingen, Germany

**Keywords:** COVID-19, parents, risk factors, protective factors, psychological burden

## Abstract

**Background:**

In the early phase of the COVID-19 pandemic, many restrictions hit people in ways never seen before. Mental wellbeing was affected and burden was high, especially for high-risk groups such as parents. However, to our knowledge no research has yet examined whether being a parent was not only a risk for psychological burden but also a way to cope with the COVID-19 pandemic.

**Methods:**

An online survey was used to collect data from 1,121 participants from April to June 2020. In addition to demographic variables, risk factors (financial burden, problems complying with COVID-19 restrictions, and pre-treatment due to mental health problems) and protective factors (emotion regulation, humor, and crisis self-efficacy) were collected. The dataset was divided into three groups: parents whose children lived at home (*n* = 395), parents whose children did not (no longer) live at home (*n* = 165), and people who were not parents (*n* = 561).

**Results:**

A linear mixed effect model showed that parents had no higher burden than non-parents, and even less when children did not live at home. Expected risk factors were generally less important, and there were no differences between parents and non-parents. In contrast, parents had advantages in protective factors.

**Conclusion:**

In the early phase of the COVID-19 pandemic, it was shown that parents (with and without their children at home) were not necessarily at risk due to additional burden, but also had prospects of coping better with the situation than people without children.

## Introduction

In March 2020, the World Health Organization declared the spread of the Corona Virus Disease 2019 (COVID-19) a global pandemic (World Health Organization, 2020). As a result, several governments responded with public health measures and restrictions to prevent the spread of the Severe-Acute-Respiratory-Syndrome-Corona-Virus-2 (SARS CoV-2). These measures included travel restrictions, social distancing and closures of workplaces, daycare centers, schools and universities. A negative influence on mental health due to the pandemic and the accompanying restrictions has been shown (Wang et al., 2020; Xiong et al., 2020; Fountoulakis et al., 2022). However, countries under consideration differed in the burden experienced, which is why no general statement can be made. Rather, a specific consideration of countries and their measures is required (Wang et al., 2021). Reasons for the observed burdens are diverse, including restrictions or limitations due to social distancing. Social distancing was found to be a risk factor for depression, generalized anxiety disorder, insomnia, and stress in the initial phase of the COVID-19 pandemic (Hoffart et al., 2020; Marroquín et al., 2020). In summary, the early phase of the COVID-19 pandemic showed a reduction in general wellbeing and an increase in affective and stress-related symptoms.

Especially in subgroups like parents, the COVID-19 pandemic seems to have a tremendous impact on wellbeing (Gamonal-Limcaoco et al., 2021; Huebener et al., 2021; Russell et al., 2021), with parents reporting more feelings of burnout and more mental health issues than non-parents (Elder and Greene, 2021; Alonzo et al., 2022).

During the pandemic, parents experienced a double burden: In addition to their own restrictions, they were also affected by those of their children, such as home schooling, the effects of their children's social distancing (Clemens et al., 2020), or the omission of previous coping strategies they engaged in as a family (Petretto et al., 2020). Overall, financial worries, lack of social support and the balance between home office, parenting, and education were very stressful burdens for parents (Chung et al., 2020; Fontanesi et al., 2020). A recent study also found that parents, whose children had home schooling, had higher psychological distress compared to parents, whose children had no home schooling, or parents who did not have school-age children (Calear et al., 2022). Additionally, an Italian study with children and adolescents showed that in particular parents with higher levels of education might be exposed to higher stress due to home schooling. The reason for this could be that parents with a higher level of education have jobs that enable remote work, which in turn has to be coordinated with home schooling (Oliva et al., 2021). Following the onset of the pandemic, parents consistently showed increased stress levels (Miller et al., 2020), with an additional study finding that over 75.0% of parents reported moderate stress levels (Susilowati and Azzasyofia, 2020). Furthermore, parents also reported an increase in negative feelings such as depression or anxiety (Brown et al., 2020; Galindo-Vázquez et al., 2020; Wu et al., 2020; Achterberg et al., 2021; Calvano et al., 2021). These psychological burdens can go even beyond a clinical threshold as the prevalence of severe mental illness was found to be 44.3% among Canadian parents with children under 18 in their own household (Gadermann et al., 2021). Similarly, a Chinese parent sample showed that burdens were lower if children were older (college students) in comparison to having younger children (other levels of children's educational status) (Wu et al., 2020). These findings imply differences depending on the care required and parenting responsibilities which in turn depend on whether children are younger or older, already in college, or no longer living at home. Thus, parents whose children live at home may be generally more at risk for increased burden due to the COVID-19 pandemic and its consequences than parents whose children no longer live at home. This burden is likely to be expressed particularly in symptoms of depression and anxiety, as previous consideration of the impact of the COVID-19 pandemic has shown (Grover et al., 2020; Wang et al., 2020). In turn, psychological distress of parents due to COVID-19 can have a negative influence on the emotional and behavioral wellbeing of their children (Dalton et al., 2020).

In addition to being a parent itself, there are other risk factors that could increase mental burden associated with the COVID-19 pandemic that have a particularly amplifying effect on parents. For many people, lockdown has been associated with reduced income, for example, in a study conducted in Vietnam, 66.9% had a reduction in their household income in the beginning of the pandemic (Tran et al., 2020). Decreased income has been shown as a risk factor for mental health (Duarte et al., 2020). In this regard, foster parents were found to be at higher risk for parental stress due to income insecurity (Miller et al., 2020). Apart from a financial constraint, more general constraints also proved to be influential. The wellbeing of parents is influenced by an increased perception of the difficulties and problems caused by the restrictions (Spinelli et al., 2020). Mental illness in parents diagnosed prior to the COVID-19 pandemic was also associated with increased depression and anxiety symptoms during the early phase of the pandemic (Wu et al., 2020). Therefore, specific consideration of financial constraints, difficulties in coping with the restrictions due to the COVID-19 pandemic, and prior treatment due to mental illness is useful to differentially assess the risk of parents, with or without children at home, compared to those who are not parents.

In contrast to the increased risk, however, there are also benefits to being a parent when it comes to mental health (Nomaguchi and Milkie, 2003). The extent to which being a parent may have had a protective effect during the early COVID-19 pandemic should therefore also be examined. Of particular interest in this context is self-efficacy in crises, to which the COVID-19 pandemic can appropriately be counted. Among other aspects, crisis self-efficacy is positively moderated by the status of being a parent (Tip et al., 2020). This may result from parents' experience that crises always occured, but could usually be overcome. In particular, feelings of control and self-efficacy have been shown to be protective during COVID-19 quarantine for parents (Brown et al., 2020; Wu et al., 2020). Especially, crisis self-efficacy, as well as emotion up-regulation strategies, showed beneficial effects in the early phase of the COVID-19 pandemic (Schelhorn et al., 2022), but with no regard to the status of being a parent. Self-care and the psychological flexibility to recognize situational demands and to react adaptively also serve as resilience factors for strengthening mental health (Coyne et al., 2020). Since emotion regulation seems to differ between parents and non-parents (Rutherford et al., 2015), emotion regulation strategies could thus have a crucial protecting influence for parents during the COVID-19 pandemic. Moreover, adaptive emotion regulation strategies for stress reduction and recovery seem to have a decisive positive influence on self-perception, partnerships, family and working life during the COVID-19 pandemic (Restubog et al., 2020). Therefore, it seems reasonable to consider the possible influence of the use of emotion regulation strategies differentiated for people with/without children. Another protective factor against stress seems to be the use of humor (Martin et al., 2003), as humor has potentially positive effects on physical and psychosocial health and wellbeing (Lefcourt, 2001; Martin, 2001). During the COVID-19 pandemic, humor was shown to transmit positive emotions (Amici, 2020), with self-enhancing humor as a style especially leading to reduced hopelessness and lower stress levels (Olah and Ford, 2021). Whether there is a difference in humor depending on parental status has not been investigated in detail as of now and is deserving of examination. It may be that parenting contributes to self-enhancing humor and thus serves as a protective factor also during the pandemic. Thus, there are several aspects that may have been protective at the onset of the pandemic and therefore are important to include in a closer look at the burden on parents and non-parents.

While several studies investigated psychological burden in parents as well as related factors increasing or decreasing burden during the COVID-19 pandemic, no study has, to our knowledge, specifically compared parents with children living at home with those whose children do not live at home and with non-parents. Additionally, the inclusion of not only risk but also protective factors of parents could shed further light on the nature of the differences between these three groups. Providing data from a German sample during the first phase of the COVID-19 pandemic also contributes to a better understanding of potential cross-country differences and similarities.

Based on the risk factors (financial burden, restrictions, previous treatment due to mental illness) outlined above, we hypothesized that parents with children living at home were more burdened due to the COVID-19 pandemic and its consequences than parents whose children do not live at home or people without children. In addition, based on the potential protective factors of parenthood (crisis self-efficacy, emotion regulation, humor), we expected parents whose children did not live at home to have a protective advantage over people without children.

## Methods

### Study design

A cross-sectional design was used to collect survey data in a web-based questionnaire. The data was collected between April 15 and June 3, 2020. General lockdown measures were introduced in Germany on 22 March 2020 and started loosening on 20 April 2020. For a simplified overview of lockdown measures across the German federal states in which restrictions varied, the restrictions of contacts (85.71% with restriction), schools (37.65% with restriction) and restaurants (84.37% with restriction) on the respective survey day were used.

### Sample

Participants were recruited *via* flyers, social online platforms, mailing lists and notices in in-patient clinics and supermarkets. The raw dataset contained 2,506 participants with usable data for statistical analysis. Inclusion criteria were: living in Germany and a sufficient understanding of German language to answer the questions. Exclusion criteria were age younger than 24 years (oriented toward the youngest parent), unrealistic or missing values as well as not living in Germany during the restrictions. The final sample contained 1,121 complete datasets (72.70% female, age range = 24–88 years, *M* = 40.33, *SD* = 13.38). The majority of participants were employed (60.57%) and it was a highly educated sample, with over 54.30% possessing at least a bachelor's degree. Participants were distributed across Germany with the majority living in Lower Saxony (25.42%) and Bavaria (22.57%). This high proportion of recruited participants in these federal states results from recruitment locations in these federal states. All participants gave their informed consent for participation and completing the questionnaires electronically. An indication of the e-mail address for a participation in follow-up measurements was voluntary. Data was collected anonymously without IP addresses or GPS tracking. This study was approved by the Ethics Committee of the Department of Psychology at the PFH Private Hochschule Göttingen (Ethics application number: 251982).

#### Subsamples

The sample was divided into 3 groups: participants whose children lived with them in the household (“parent with child at home,” *n* = 395), participants whose children no longer lived with them in the household (“parent without child at home,” *n* = 165) and participants without children (“no parent,” *n* = 561). A demographic overview of each group is depicted in Table 1. Some descriptive characteristics can be identified here. The group “no parents” had a higher proportion of bachelor's degrees in education, a higher proportion of students and a younger average age. On the other hand, the group “parent without child at home” is the group with the oldest average age, the lowest education level and the highest proportion of retired participants. Thus, it seems that the groups not only differ in whether they have children (at home), but are also in other stages of life. Thus, controlling for these variables seems to be of great relevance.

**Table 1:** Demographic characteristics of subsamples.

	**Groups**		
**Variable**	**Parents with children at home**	**Parents without children at home**	**No parents**
**Age**			
*M* (*SD*)	44.00 (9.81)	58.68 (9.49)	32.35 (9.55)
Range	24–82	33–82	24–88
**Gender** ***n*** **(%)**			
Male	88 (22.28)	59 (35.76)	159 (28.34)
Female	307 (77.72)	106 (64.24)	402 (71.66)
**Education** ***n*** **(%)**			
Lower Secondary School	4 (1.01)	6 (3.64)	2 (0.36)
GCSE	24 (6.08)	23 (13.94)	24 (4.28)
Vocational Training	89 (22.53)	43 (26.06)	113 (20.14)
GCE	63 (15.95)	26 (15.76)	94 (16.76)
Bachelor's degree	50 (12.66)	21 (12.73)	167 (29.77)
Master's degree	154 (38.99)	43 (26.06)	151 (26.92)
Doctoral degree	10 (2.53)	3 (1.82)	8 (1.43)
**Children**			
*M* (*SD*)	2.06 (0.95)	2.16 (1.40)	–
Range	1–6	1–10	
**Employment status** ***n*** **(%)**			
Employed	233 (58.99)	83 (50.30)	363 (64.71)
Self-employed	80 (20.25)	24 (14.55)	51 (9.09)
Civil servant	42 (10.63)	15 (9.09)	43 (7.66)
Student / Trainee	30 (7.59)	3 (1.82)	149 (26.56)
Unemployed	17 (4.30)	7 (4.24)	11 (1.96)
Retired	9 (2.28)	45 (27.27)	15 (2.67)
Maternity protection, Parental leave	27 (6.84)	–	1 (0.18)

GCSE, General Certificate of Secondary Education; GCE, General Certificate of Education. In education, n = 1 misssing for group “parents with child at home” and n = 2 missing for group “no parents.” Multiple choices possible in employment status.

### Measures

#### Psychological burden

Psychological burden was assessed with the self-report questionnaire ICD-10-Symptom-Rating (ISR; Tritt et al., 2008). The ISR is used as a diagnostic screening instrument in German-speaking countries. In total, the ISR contains 29 items based on the diagnostic criteria of the ICD-10 (Tritt et al., 2008). The five symptom subscales depression (example item: “W*hen I want to do something I lack energy and get tired quickly*”), anxiety (“*Just thinking about a possible anxiety attack scares me*”), eating disorder (“*I spend a lot of time thinking of ways to lose weight*”), obsessive-compulsive disorder (“*I try to resist reoccurring, seemingly senseless thoughts and actions, but often don't succeed*”), and somatoform disorder (“*I worry about having a serious physical illness*”) are formed from 3 to 4 items each, 12 additional items are used to screen for individual syndromes such as derealization. All items were rated on a 5-point Likert-scale from 0 to 4, with 0 indicating “*does not apply*” and 4 indicating “*applies extremely*.” The item scores are averaged for each subscale and the subscales can be added to form a total score. The period surveyed is the last 2 weeks, which is an optimal period considering pre-existing restrictions. The reported internal consistency for the total score is very good (Cronbach's α = 0.92), with slightly lower but still good consistency for the subscales (Cronbach's α = 0.78–0.86). The reliability of the ISR is satisfactory (Fischer et al., 2011). A strong association (*r* = 0.84) between the total score of the ISR and the Global Severity Index of the Symptom-Checklist-90-R (SCL-90 R; (Franke, 2002)) also confirmed the validity of the ISR (Tritt et al., 2010). Strengths of the ISR are its brevity and its pragmatic approach to good scientific quality criteria.

Possible protective factors against negative effects of COVID-19 risk and associated restrictions were assessed by the three constructs: emotion regulation, humor, and crisis self-efficacy.

#### Emotion regulation

Due to the lack of instruments for the simple measurement of emotion regulation strategies, a self-constructed questionnaire with 8 items was used to assess up-regulation of pleasant emotions. Construction was based on the process model of emotion regulation by Gross (2014). In particular, strategies present already “before the event,” and formulations introduced by Quoidbach et al. (2010) for savoring strategies were used. Accordingly, half of the items addressed savoring of pleasant emotions, the other half addressed up-regulation of pleasant emotion through pre-event strategies. All items were set in relation to the pandemic and subsequent restrictions and had to be answered on a 5-point Likert-scale from “*does not apply*” to “*applies extremely*.” The answers were averaged for further analyses. The internal consistency of the used items was very good in our sample, with Cronbach's α = 0.92.

#### Humor

The potential positive influence of humor as an adaptive coping strategy was assessed using the subscale “self-enhancing humor” of the Humor Styles Questionnaire by Martin et al. (2003). This distinct humor style is measured *via* an 8-item subscale, of which 2 items were not used due to bad fit with the context of the restrictions (“…amusing aspect of a situation…” and “I don't need to be with other people…”). Answers were made on a 7-point Likert-scale, ranging from “*totally disagree*” to “*totally agree*.” The items were averaged to a score. Self-enhancing humor was found to be negatively associated with depression and anxiety (Martin et al., 2003; Kuiper et al., 2004). For the German version, internal consistency for this subscale was found to be good with Cronbach's α = 0.83, factorial validity was also confirmed (Ruch and Heintz, 2016). Internal consistency in our sample was good, Cronbach's α = 0.80.

#### Crisis self-efficacy

To assess the possible influence of participants' belief of self-efficacy, a translated extract of the crisis self-efficacy index (Park and Avery, 2019) was used. This was done using 4 of the original 12 items, one of each of the 4 factor fractures that had the highest load on the factor. The items were answered on a 7-point Likert-scale, from “*strong disapproval*” to “*strong approval*.” Thereby, the four factors of the index (action, preventive, achievement, and uncertainty management) could be assessed shortly and combined into an average score. In our sample, the used items showed low intercorrelations, suggesting a good divergence between the items. Internal consistency was acceptable for our sample, with Cronbach's α = 0.69.

### Statistical analysis

Statistical analyses were conducted using the R statistical package, version 4.0.2 (R Core Team, 2021) for linear mixed effect models (LMEs), and SPSS 28 (IBM Corp Released, 2021) otherwise. In order to assess which factors contributed to an increased ISR sum score during the early phase of COVID-19 pandemic, LMEs were computed through the *lme4* package in R (Bates et al., 2015). LMEs have several advantages compared to other means of analyses, among them robustness with unequal sample sizes and missing data, and non-normally distributed dependent variables (Judd et al., 2017). Furthermore, LMEs can include random effects and assess additional variability where some groups have fewer entries than others (i.e., in education). *P*-values were computed *via t*-tests using the Satterthwaite approximation to degrees of freedom *via* the package *lmerTest* (Kuznetsova et al., 2017). Additionally, the package *r2glmm* (Jaeger et al., 2017) aided in determining *R*^2^ for fixed effects including confidence intervals for effect sizes.

In a first step, the ISR sum score was used as a dependent variable, followed by models with the ISR depression score and the ISR anxiety score as dependent variables. Independent variables were chosen based on bi-variate correlations (Kendall's τ) between the outcome measure and the covariates humor, emotion regulation strategy, crisis self-efficacy, and age. Factors were chosen according to the criteria (a) relevance to mental health, (b) relevance during the COVID-19 pandemic and (c) being descriptive of the population. Additionally, Mann-Whitney *U* tests (2 categories) and Kruskal–Wallis *H* tests (3 and more categories) were computed to determine significant differences in the outcome variables depending on categories. Factors with more than two categories were recoded as dummy variables. For group assignment, being a parent with child at home was chosen as the reference category.

In order to calculate effect sizes for individual model terms, the semi-partial (marginal) *R*^2^ by Jaeger et al. (2017) is reported in addition to confidence intervals. Effect sizes of 0.14 are interpreted as large effects, 0.06 as medium effects, and 0.01 as small effects. All models followed best practice recommendations for model-fitting (Barr, 2013), beginning with a null model including a random intercept which is then compared to a maximized model. In a final step, a reduced model was computed and compared to both the null and the maximized model. Model indices were the Akaike Information Criterion (AIC: Akaike, 1974), the Bayesian Information Criterion (BIC: Schwarz, 1978) and the log likelihood ratio (LR) statistic. In the end, the model with the lowest AIC and BIC values that was significantly different from the null model was chosen. The general modeling strategy was the following:


(1) $$\begin{array}{l} \text{ISR\_Score }\sim \text{ age + sex + no parent + parent no child}\\ \text{ + reduction in income + COVID compliance}\\ \text{ + humor + emotion strategy + crisis efficacy}\\ \text{ + psych treatment + (1|education)} \end{array}$$

Where *no parent* refers to the group of participants that are not parents, *parent no child* refers to the group of participants that are parents but where children are not living at home; *reduction in income* refers to reduced income during the lockdown; *COVID compliance* refers to difficulties complying with the COVID-19 restrictions; *humor* refers to the self-enhancing humor score; *emotion strategy* refers to the emotion strategy total score; *crisis efficacy* refers to the crisis self-efficacy score; and *psych treatment* refers to whether participants have been in treatment for a psychiatric disorder before. Treatment was defined as ambulant therapy for a duration of at least three months or stationary therapy for at least two weeks. As a final step, education was added as a random effect as comparable values were expected within educational groups. Additionally, group differences in protective variables (crisis self-efficacy score, self-enhancing humor score and emotion regulation score) were computed using Kruskal–Wallis tests, followed by *post-hoc* tests. Multiple comparisons were corrected for using the False Discovery Rate (FDR) correction (Benjamini and Hochberg, 1995) and the *p*-value was set to 0.05.

## Results

In the examined sample, more than half of the participants showed inconspicuous total ISR values, but several participants scored above clinical cut-offs. Non-parents had the highest percentage with low to severe symptom burden in the total ISR score (40.50%, *n* = 226), followed by parents with children at home (30.79%, *n* = 121) and finally parents without children at home with the lowest values (27.44%, *n* = 45).

In order to determine independent variables suitable for LMEs predicting ISR scores, bi-variate correlations were computed (see Table 2). The ISR sum score was significantly correlated with age (τ = −0.14, *p* < 0.001), COVID compliance (τ = 0.17, *p* < 0.001), humor (τ = −0.13, *p* < 0.001), emotion strategy (τ = 0.13, *p* < 0.001) and crisis efficacy (τ = −0.25, *p* < 0.001). Identical patterns could be observed for the specific ISR scores for depression and anxiety. For categorical variables, significant differences in the ISR sum score could be observed for the variables sex (*U* = 96468.00, *z* = −3.67, *p* < 0.001), income (*H*_(2)_ = 13.27, *p* < 0.001), group (*H*_(2)_ = 10.78, *p* = 0.005) and psychological treatment (*U* = 43550.50, *z* = −9.80, *p* < 0.001). Significant differences in the ISR subscale depression were found for income (*H*_(2)_ = 15.58, *p* < 0.001), sex (*U* = 99738.50, *z* = −2.97, *p* = 0.003), group (*H*_(2)_ = 10.81, *p* = 0.004), and psychological treatment (*U* = 49879.50, *z* = −8.19, *p* < 0.001). For the ISR variable anxiety, differences were observed for income (*H*_(2)_ = 10.02, *p* = 0.007), sex (*U* = 94589.50, *z* = −3.54, *p* < 0.001), and psychological treatment (*U* = 56282.50, *z* = −7.23, *p* < 0.001). Accordingly, these variables were added into the LMEs for the respective ISR variable.

**Table 2:** Correlations between LME variables.

	**ISR depression**	**ISR anxiety**	**Age**	**Difficulties with restriction compliance**	**Self-enhancing humor**	**Emotion regulation**	**Crisis self-efficacy**
ISR sum score	0.651^*^	0.583^*^	−0.142^*^	0.173^*^	−0.127^*^	0.134^*^	−0.248^*^
ISR depression		0.417^*^	−0.134^*^	0.263^*^	−0.174^*^	0.073^*^	−0.261^*^
ISR anxiety			−0.105^*^	0.075^*^	−0.098^*^	0.132^*^	−0.241^*^
Age				0.003	0.069^*^	−0.068^*^	0.070^*^
Difficulties with restriction compliance					−0.067^*^	0.039	−0.087^*^
Self-enhancing humor						0.197^*^	0.211^*^
Emotion regulation							0.019

* p < 0.001.

### Linear mixed effect models

LMEs were fit with the total ISR score and the specific scores for depression and anxiety as dependent variables and correlated metric variables and significant categorical variables as possible predictors. Education was added as a random effect in every LME. The null model was compared to a maximized model including all relevant predictors, followed by a reduced model only including significant predictors. This was done in order to achieve a parsimonious model. Model comparisons for all 3 LMEs are depicted in Table 3.

**Table 3:** Model comparisons of linear-mixed effect models predicting ISR scores.

**Outcome variable**	**Model**	**AIC**	**BIC**	**logLik**	***R*** ^**2**^	**vs. null model**		**vs. max model**	
						**χ^2^**	***p*** **-value**	**χ^2^**	***p*** **-value**
ISR sum score	Null Model	1886.70	1901.60	−940.40	0.00				
	Max Model	1552.90	1622.50	−762.44	0.28	355.84	<0.001		
	Reduced Model	1548.00	1602.7	−763.01	0.28	354.71	<0.001	1.13	0.768
ISR depression	Null Model	2803.10	2818.00	−1398.50	0.00				
	Max Model	2391.8	2456.50	−1182.90	0.34	431.24	<0.001		
	Reduced Model	2387.30	2447.0	−1181.70	0.34	428.23	<0.001	3.00	0.391
ISR anxiety	Null Model	2679.92	2694.84	−1337.00	0.00				
	Max Model	2449.30	2504.00	−1213.60	0.21	246.65	<0.001		
	Reduced Model	2452.90	2487.80	−1219.50	0.20	234.99	<0.001	11.65	0.020

ISR = ICD-10-Symptom-Rating. The null model refers to a random-slope-only model. The max model refers to a model including all predictors and the reduced model only includes significant predictors from the max model.

With the exception of the ISR anxiety score, the reduced model was to be preferred over the maximized model. Significant predictors for the ISR sum score were age, COVID compliance, being a parent without children at home, reduction in finances, the self-enhancing humor score, the emotion regulation strategy score, crisis self-efficacy score and prior psychological treatment. Significant predictors for the ISR depression score were a reduction in finances, COVID compliance, the self-enhancing humor score, the emotion regulation strategy score, the crisis self-efficacy score and prior psychological treatment. Finally, for the ISR anxiety score, significant predictors were age, the emotion regulation strategy score, the crisis self-efficacy score and prior psychological treatment. The respective LMEs and predictors are depicted in Table 4.

**Table 4:** Main fixed effects of interest within LMEs predicting ISR scores.

	**Fixed effect**	**Estimate**	***SD***	***t***	***p***	$R_{\beta^{*}}^{2}$	$R_{\beta^{*}}^{2}$ CI
ISR sum score	Age	−0.01	0.00	−3.65	<0.001	0.01	0.00–0.03
	Difficulties with COVID-19 compliance	0.09	0.02	5.28	<0.001	0.03	0.01–0.05
	Parent, kid not at home	−0.06	0.03	−2.17	0.030	0.00	0.00–0.02
	Reduction in income	−0.04	0.02	−2.14	0.032	0.00	0.00–0.02
	Self-enhancing-humor	−0.01	0.00	−2.70	0.007	0.01	0.00–0.02
	Emotion regulation strategy	0.01	0.00	5.98	<0.001	0.03	0.02–0.06
	Crisis self-efficacy	−0.05	0.00	−11.85	<0.001	0.12	0.08–0.15
	Previous psychological treatment	−0.13	0.02	−6.47	<0.001	0.04	0.02–0.06
ISR depression	Age	−0.01	0.00	−4.04	<0.001	0.02	0.00–0.03
	Reduction in income	−0.06	0.03	−2.47	0.014	0.01	0.00–0.02
	Difficulties with COVID-19 compliance	0.26	0.03	9.93	<0.001	0.09	0.06–0.12
	Self-enhancing-humor	−0.02	0.00	−3.47	<0.001	0.01	0.00–0.03
	Emotion regulation strategy	0.01	0.00	3.75	<0.001	0.01	0.00–0.03
	Crisis self-efficacy	−0.07	0.01	−11.71	<0.001	0.11	0.08–0.15
	Previous psychological treatment	−0.21	0.03	−6.63	<0.001	0.04	0.02–0.07
ISR anxiety	Age	−0.00	0.00	−2.51	0.012	0.01	0.00–0.02
	Sex	−0.04	0.03	−1.50	0.133	0.00	0.00–01
	Reduction in income	−0.04	0.03	−1.50	0.133	0.00	0.00–0.01
	Difficulties with COVID-19 compliance	0.05	0.03	1.95	0.051	0.00	0.00–0.01
	Self-enhancing-humor	−0.01	0.00	−1.59	0.113	0.00	0.00–0.01
	Emotion regulation strategy	0.02	0.00	4.67	<0.001	0.02	0.01–0.04
	Crisis self-efficacy	−0.06	0.01	−10.59	<0.001	0.09	0.06–0.13
	Previous psychological treatment	−0.16	0.03	−5.08	<0.001	0.02	0.01–0.04

LME, Linear Mixed Effect Model; ISR, ICD-10-Symptom-Rating; CI, Confidence Interval.

**Figure 1:**
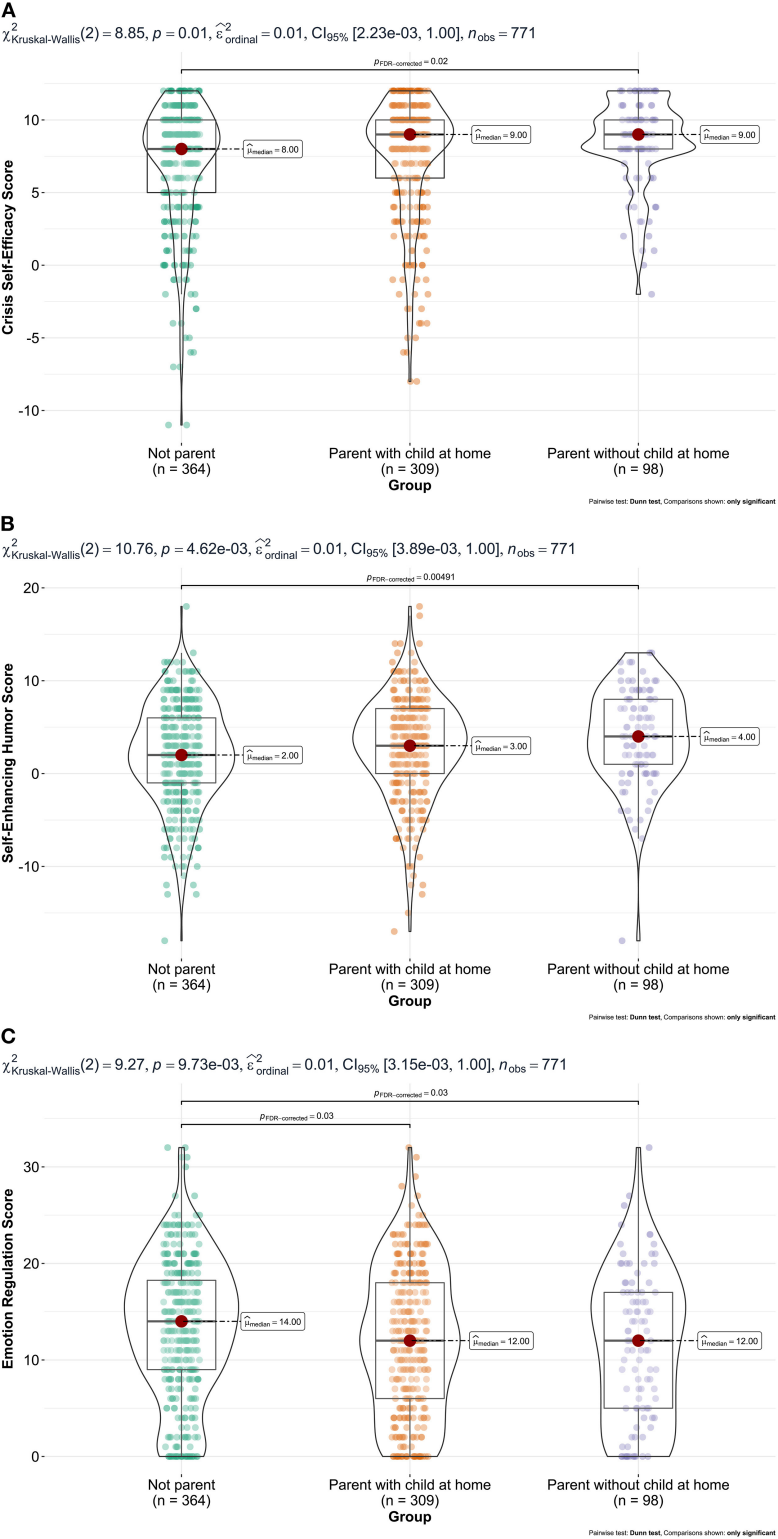
Group comparisons for **(A)** crisis self-efficacy, **(B)** self-enhancing humor, and **(C)** emotion regulation.

As the crisis self-efficacy score, the self-enhancing humor score and the emotion regulation strategy score played an important role for the prediction of the ISR, group differences in the respective scores were computed using Kruskal–Wallis tests, followed up by *post-hoc* tests. See Figure 1 for a detailed depiction of the group comparisons.

## Discussion

The aim of this study was to investigate whether parents show greater psychological burden associated with the early COVID-19 pandemic and its counter measures, than people without children (at home). The psychological burden of parents with children at home was considered in relation to possible risks (financial burden, problems with COVID-19 restrictions, and previous psychological treatment) and protective factors (emotion regulation, humor, crisis self-efficacy) and compared to people without children and parents whose children no longer live at home.

Our model showed that the status of “being a parent” is neither a risk nor protective in general but has to be considered differentially whether children live at home or not. It can be assumed that the majority of children who live at home are also younger and therefore need more supervision and care. Accordingly, our model shows that “being a parent” is negatively associated with psychological burden when children are not at home compared to when they are. This is in line with previous findings, suggesting that children who no longer live at home may play a supporting role for their parents (Wu et al., 2020). There were no differences between people who are not parents and parents with children at home, which was unexpected. This may be due to the heightened burden of young people in the early phase of the COVID-19 pandemic (Glowacz and Schmits, 2020; Hawes et al., 2021) considering on average people without children were younger in our sample. Furthermore, this negative predictor only accounted for the total burden score, not in the specific areas of depression or anxiety. Thus, being a parent seems not to be associated with a greater risk for psychological burden due to the early phase of the COVID-19 pandemic in comparison to not being a parent, whereas being a parent without children at home represented a protective factor.

Age was found to be a weak negative predictor; higher age was associated with a lower total ISR score, as well as with lower depression and anxiety scores. Relating to resilience, age has been shown to have a positive age-affect relationship (Mroczek and Kolarz, 1998) which could have been protective. Besides, emotional wellbeing tends to increase with age (Charles and Carstensen, 2010). Therefore, it seems reasonable that the impact of the early phase of the COVID-19 pandemic on psychological burden was negatively associated with increasing age. An analysis of data from a community sample also showed that middle-aged adults were less mentally burdened than young adults at the beginning of the COVID-19 pandemic (Pothisiri and Vicerra, 2021).

Sex was not a relevant predictor of overall psychological burden, with the exception of anxiety symptoms. In the present study, a negative relationship between male sex and psychological burden could be found, with men having lower anxiety symptoms than women. This is in line with previous research during the COVID-19 pandemic (Galindo-Vázquez et al., 2020; Luceño-Moreno et al., 2020).

Looking at potential risk factors (i.e., financial burdens due to the COVID-19 pandemic and its restrictions, difficulties in compliance with the restrictions due to COVID-19 or previous psychological treatment), a rather mixed pattern emerged: A reduction in income was shown to be a positive predictor of psychological distress—overall, as well as specifically for depression and anxiety. *Post*-*hoc* analyses showed no differences between the three groups. However, this counterintuitive direction may be due to several factors. First, a majority of participants had either less or unchanged income due to the COVID-19 crisis in our survey. Thus, an increase in income cannot be interpreted here in relation to psychological burden. Second, the effect of experiencing less psychological burden with less income might be explained *via* German government programs. Thus, employment and a majority of previous income was secured, even though individuals were not able to engage in employment for a certain period of time. This may have had a relieving effect at the beginning of the pandemic which fits in with the relevant depression and anxiety domains. Though it seems not possible to estimate the long-term effect of the reported financial changes, a study in Germany conducting data a few weeks after the end of this survey (July and August 2020) did indeed show a higher burden in depressive symptoms among students with reduced income (Kohls et al., 2021).

Further, difficulties in compliance with restrictions due to the COVID-19 pandemic was a positive predictor of psychological burden. *Post-hoc* analyses showed no differences between the three groups. People who suffered from restrictions such as social distancing or closed shops also had higher scores in total psychological burden, depression and anxiety. Accordingly, a study from Hong Kong at the beginning of the COVID-19 pandemic also showed that problems in compliance with COVID-19 restrictions were associated with higher depression and anxiety scores (Zhao et al., 2020).

Previous psychological treatment was shown to be a negative predictor of psychological burden overall and in particular for depression and anxiety domains during the early COVID-19 pandemic. *Post-hoc* analyses identified no differences between the three groups. Thus, earlier psychological treatment was a protective factor rather than a risk with no differences for (non) parents. Earlier treatment may have helped to develop coping strategies which were also helpful during the pandemic. Nevertheless, this statement refers only to previous psychiatric pretreatment. Individuals with existing mental illness at the COVID-19 outbreak are considered a high-risk group (Neelam et al., 2021) and should therefore be monitored with appropriate attention.

In addition to potential risks, potential protective factors (i.e., emotion regulation, self-enhancing humor, and crisis self-efficacy) and their potential differential effects on the different groups were also assessed.

The use of emotion regulation in the sense of up-regulation of positive emotions was found to be a positive predictor with a very small effect on psychological burden (total score as well as depression and anxiety). That the ability and use of an adaptive emotion regulation strategy is associated with higher psychological burden is unexpected and contrary to previous findings for adaptive cognitive emotion regulation strategies and wellbeing (Gubler et al., 2020). It is conceivable that particularly burdened people first have to resort to this special strategy, whereas less burdened people can still manage without applying such a strategy. Interestingly, *post-hoc* analyses showed that parents with children at home used less up-regulation of positive emotions than people without children. Accordingly, Gambin et al. (2020) were able to show that difficulties in emotion regulation are not predictive of positive experiences in the parent-child relationship. There was no difference between parents with and without children at home.

As hypothesized, adaptive humor, especially self-enhancing humor, emerged as a negative, although weak predictor in our model. People with the tendency to handle situations humorously showed less psychological burden than others. This is in line with previous findings regarding the COVID-19 pandemic (Amici, 2020). *Post-hoc* analyses revealed that parents whose children do not live at home (anymore) show a higher score of self-enhancing humor than people without children. Since our data are cross-sectional, it is not possible to conclude whether these differences are based on humor development through parenting or not. In any case, humor seems to have been a beneficial skill to cope with the burdens of the early COVID-19 pandemic.

As expected, crisis self-efficacy, as a measure that particularly applies in difficult, unusual situations, turned out to be a negative predictor in our model. A higher conviction of being able to overcome crises under own effort was thus associated with a lower value of psychological burden. In detail, parents whose children do not live at home (anymore) showed the highest score, followed by parents whose children live at home, while people without children having the lowest score. Unlike humor, there is certainly evidence of parents showing higher scores on crisis self-efficacy (Tip et al., 2020). This may also suggest that parents whose children do not live at home (anymore) have experienced this development for a longer period of time and thus have higher scores than parents whose children still live at home.

### Limitations

Several limitations of the study should be mentioned: First, about half of the data presented cover only 2 of the 16 federal states of Germany, i.e., Bavaria and Lower Saxony. This was due to increased recruitment in these two locations and should not have a significant impact on generalizability across Germany. In line with this, Schelhorn et al. (2022) did not show significant differences in psychological burden between the two states in an analysis of the same data used here. Second, the categorization into the three groups (parents with children at home, parents without children at home, and non-parents) omits information such as the number and age of children in the household, which could contribute to a more differentiated interpretation of risks and resilience within the respective groups of parents. However, data accessed in this study provide a first overview of the parental situation in regard to their psychological wellbeing during the early phase of the COVID-19 pandemic. In future studies, a more detailed analysis of these different groups of parents is clearly needed, as is a specification of potentially useful information regarding the children. For instance, it is not possible to say whether, in the case of parents whose children are not at home, the children have moved out to set up their own household or, for example, had to be temporarily placed in a care home. We consider this to be a negligible exception, however, it should be taken into account when interpreting the results. Third, the examined protective factors are based on scales that have been shortened (crisis self-efficacy), are a situationally appropriate shortened subscale of a questionnaire (self-enhancing humor), or are, theory-driven, self-generated (emotion regulation strategy). This limitation derives from the need for a survey that is comprehensive but short in order to gather as much information as possible in a low-threshold manner. Despite the mentioned adaptations, the scales in this sample proved to be reliable (Cronbach's α = 0.69–0.92) and therefore very profitable. Fourth, since the sample was intended to represent as broad a picture of the community as possible, mental health diagnoses were neglected in general, and only severe mental distress with therapy attendance was recorded. On the one hand, this could have led to a certain proportion of mentally ill people among the participants, which must be taken into account when interpreting the ISR values. On the other hand, this in turn contributes to the improved generalizability of the results, since mental illnesses are part of society. Finally, it must be mentioned that this study is a cross-sectional excerpt. The insights obtained and predictors named can therefore only be interpreted with caution; no causal relationships can be derived. For causal interpretations, repeated measurements in the pandemic, distributed over a larger period of time, would be of particular value. Nevertheless, an overview of the investigated constructs during the first period of the COVID-19 pandemic is of particular importance due to the partly chaotic and unfamiliar nature of this period.

## Conclusion

The present results on the early phase of the COVID-19 pandemic examining psychological burden in Germany show that people were also able to access a number of protective factors. Parents in particular had advantages in addition to their specific risk factors, showing higher resilience in the areas of humor and crisis self-efficacy, and having to resort less to emotion regulation strategies compared to non-parents. In addition, the results generally reveal that, out of the expected risks, only difficulties in complying with COVID-19 restrictions were relevant, while financial burdens or previous mental health treatment were associated with lower psychological distress. Thus, being a parent is not a risk factor by itself, but must be considered differentially.

## Data availability statement

The raw data supporting the conclusions of this article will be made available by the corresponding author on request without undue reservation.

## Ethics statement

The studies involving human participants were reviewed and approved by Ethics Committee of the Department of Psychology at the PFH Private University of Applied Science, Göttingen (Ethics application number: 251982). The patients/participants provided their written informed consent to participate in this study.

## Author contributions

Conceptualization and validation: AE, IJ, DS, IS, and YS. Data curation: AE, IJ, and YS. Formal analysis: IJ. Funding acquisition and resources: YS. Investigation: AE, IS, and YS. Methodology: AE, IJ, DS, IS, MM, TB, and YS. Project administration: AE. Software: ML and IJ. Visualization: AE and IJ. Supervision: SK. Writing—original draft: AE, IJ, and DS. Writing—review and editing: SK, MM, and TB. All authors contributed to the article and approved the submitted version.

## Funding

This study was part of a larger project funded by Inland Norway University of Applied Sciences.

## Conflict of interest

The authors declare that the research was conducted in the absence of any commercial or financial relationships that could be construed as a potential conflict of interest.

## Publisher's note

All claims expressed in this article are solely those of the authors and do not necessarily represent those of their affiliated organizations, or those of the publisher, the editors and the reviewers. Any product that may be evaluated in this article, or claim that may be made by its manufacturer, is not guaranteed or endorsed by the publisher.
